# Neighborhood Disadvantage and Cognitive Function: The Moderating Role of Self-reported Loneliness

**DOI:** 10.1093/geroni/igaf122.3290

**Published:** 2025-12-31

**Authors:** Regina Wright, Desiree Bygrave

**Affiliations:** University of Delaware, Newark, Delaware, United States; Benedict College, Columbia, South Carolina, United States

## Abstract

Neighborhood disadvantage has been linked to worse cognitive performance and greater cognitive decline among older adults; however, moderators of these associations have not been well studied. Loneliness is a potential moderator of importance—one that also worsens cognitive functioning. Given the documented influence of neighborhood disadvantage and loneliness on cognitive functioning, it is important to understand whether self-reported loneliness exacerbates this relationship. Therefore, the objective of the study was to examine associations between neighborhood disadvantage and cognitive function, and to examine whether associations are moderated by self-reported loneliness. The analysis included 136 older adults (36% male) with a mean age of 68.04y. Neighborhood disadvantage was assessed with the Area Deprivation Index (ADI), that provided state and national rankings of neighborhood deprivation. Cognitive function was assessed with the Verbal Fluency Test (verbal fluency), the Visual Reproductions Test (short- and long-term nonverbal memory), Logical Memory Test (verbal memory) and Digit Span Forward and Backward (working memory). Self-reported loneliness was measured with the UCLA Loneliness Scale. Multivariable regression analyses were run, adjusted for age, sex, and educational attainment. Results showed that greater national ADI rankings (more disadvantage) were associated with worse short-term nonverbal memory. Similarly, greater state and national ADI rankings were associated with worse long-term nonverbal memory. Additional results showed that loneliness moderated the relationship between national ADI rankings and both types of nonverbal memory. Overall, our findings suggest that greater neighborhood disadvantage may influence select domains of memory, and that one’s self-reported loneliness may influence how this relationship manifests.

